# Insulin/IGF-1 and Hypoxia Signaling Act in Concert to Regulate Iron Homeostasis in *Caenorhabditis elegans*

**DOI:** 10.1371/journal.pgen.1002498

**Published:** 2012-03-01

**Authors:** Daniel Ackerman, David Gems

**Affiliations:** Institute of Healthy Aging and Department of Genetics Evolution and Environment, University College London, London, United Kingdom; University of California San Francisco, United States of America

## Abstract

Iron plays an essential role in many biological processes, but also catalyzes the formation of reactive oxygen species (ROS), which can cause molecular damage. Iron homeostasis is therefore a critical determinant of fitness. In *Caenorhabditis elegans*, insulin/IGF-1 signaling (IIS) promotes growth and reproduction but limits stress resistance and lifespan through inactivation of the DAF-16/FoxO transcription factor (TF). We report that long-lived *daf-2* insulin/IGF-1 receptor mutants show a *daf-16*–dependent increase in expression of *ftn-1*, which encodes the iron storage protein H-ferritin. To better understand the regulation of iron homeostasis, we performed a TF–limited genetic screen for factors influencing *ftn-1* gene expression. The screen identified the heat-shock TF *hsf-1*, the MAD bHLH TF *mdl-1*, and the putative histone acetyl transferase *ada-2* as activators of *ftn-1* expression. It also revealed that the HIFα homolog *hif-1* and its binding partner *aha-1* (HIFβ) are potent repressors of *ftn-1* expression. *ftn-1* expression is induced by exposure to iron, and we found that *hif-1* was required for this induction. In addition, we found that the prolyl hydroxylase EGL-9, which represses HIF-1 via the von Hippel-Lindau tumor suppressor VHL-1, can also act antagonistically to VHL-1 in regulating *ftn-1*. This suggests a novel mechanism for HIF target gene regulation by these evolutionarily conserved and clinically important hydroxylases. Our findings imply that the IIS and HIF pathways act together to regulate iron homeostasis in *C. elegans*. We suggest that IIS/DAF-16 regulation of *ftn-1* modulates a trade-off between growth and stress resistance, as elevated iron availability supports growth but also increases ROS production.

## Author Summary

Iron plays a role in many biological processes, including energy generation and DNA replication. But to maintain health, levels of cellular iron must be just right: too much or too little iron can cause illnesses, such as anemia and hemochromatosis, respectively. Animals therefore carefully control their iron levels by regulating of iron uptake, transport, and storage within protein capsules called ferritins. But how do they coordinate this? Using the model organism *C. elegans*, we have discovered a network of genes and pathways that control iron homeostasis. We find that ferritin is regulated by insulin/IGF-1 signaling, which also controls growth and resistance to oxidative stress in response to harsh environmental conditions. Ferritin is also regulated by the hypoxia signaling pathway, which responds to oxygen and iron levels as well as to metabolic cues. We find that the hypoxia pathway acts as an iron sensor, a role it may also play in humans. Our work defines a network of signaling pathways that can adjust iron availability in response to a range of environmental cues. Understanding this network in *C. elegans* can help us to understand the causes of iron dyshomeostasis in humans, which can profoundly affect health.

## Introduction

In order to survive in a changing environment, organisms have evolved abilities to sense their surroundings and adaptively adjust their physiology. For example, the nematode *Caenorhabditis elegans* is capable of postponing reproduction if conditions are unsuitable for growth and reproduction by forming dauer larvae [1], [2], [3]. This developmentally arrested third larval stage is resistant to starvation and other stressors, allowing the animal to survive until conditions improve. Should this occur, dauer larvae can re-enter the normal reproductive life cycle.

The decision between reproductive growth and survival with enhanced stress resistance is controlled by a complex sensory/signaling network that includes the insulin/IGF-1 signaling (IIS) pathway [2]. Mutants with reduced IIS exhibit constitutive dauer larva formation, but can also form adults that are resistant to a range of stressors, including reactive oxygen species (ROS), UV irradiation, heat stress and ER stress [4], [5], [6]. IIS controls this response through the DAF-16/FoxO transcription factor, which enters the nucleus under adverse conditions and affects gene regulation [7], [8]. DAF-16 promotes increased expression of many genes encoding proteins that protect against stress, including superoxide dismutases, drug metabolizing enzymes and molecular chaperones 9,10. DAF-16 is also required for the longevity of IIS mutants, for example those with defects in the DAF-2 insulin/IGF-1 receptor [11]. Both of these roles of DAF-16, the promotion of stress resistance and longevity, will improve the chances of living through periods of adversity. Whether the same downstream mechanisms cause increased stress protection and longevity remains unclear [12].

One factor contributing to growth and stress resistance is cellular iron availability. Free intracellular iron is toxic to the cell due to its role in catalyzing the Fenton reaction, which generates hydroxyl radicals from hydrogen peroxide:
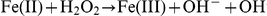
However, while free intracellular iron is harmful to the cell, iron is also an important element for a large number of cellular processes, including electron transport, deoxyribonucleotide synthesis, cellular detoxification, the cell cycle, oxygen transport and many others [13], [14]. Lack of iron is thought to affect the health of up to a billion people worldwide [15].

As well as nutritional iron deficiency, disruption of mechanisms that regulate iron homeostasis can also lead to a number of serious diseases in humans, such as anemias and iron overload disorders [16], [17]. The maintenance of appropriate iron levels is therefore important to viability and is tightly regulated by a number of proteins. These include ferritins, which form 24-subunit spherical nanocages that are each able to safely store up to 4500 atoms of iron. Heavy chain ferritins (H-ferritins) contain a ferroxidase centre, which has the capacity to convert Fe(II) to Fe(III) when the iron atom enters the complex [18].

The *C. elegans* genome contains two H-ferritin genes, *ftn-1* and *ftn-2* [19]. *ftn-1* is predominantly expressed in the intestine, while *ftn-2* is expressed in many cell types [19], [20]. In vertebrates, regulation of ferritin gene expression in response to iron levels is achieved both transcriptionally [21], and post-transcriptionally by the actions of iron regulatory proteins (IRPs) which bind to iron responsive elements (IREs) in the 5′ UTR of ferritin mRNAs [22]. Expression of *C. elegans* ferritin genes is also sensitive to iron levels: iron supplementation increases *ftn-1* expression, while iron chelation has the opposite effect. However, *ftn-1* and *ftn-2* lack IRE sequences in their 5′ UTRs and iron-dependent regulation seems to be achieved solely through transcriptional regulation [23]. The mechanism by which this occurs remains unknown, but iron-dependent regulation of *ftn-1* requires a 63 bp iron-dependent element (IDE) in its promoter [20].

Research on the regulation of *ftn-1* in *C. elegans* has contributed to our understanding of ‘restless leg syndrome’, a human disease linked to iron deficiency in the brain. A haplotype of the gene MEIS1 has been associated with inheritance of the syndrome [24] but the gene's function was unknown. The involvement of the *C. elegans* ortholog *unc-62* in regulating iron homeostasis was tested and a repressive role for this gene in *ftn-1* regulation was identified. This regulation may be conserved in humans, since reduced MEIS1 expression seems to cause increased expression of human ferritin as well as of an iron transporter [25]. Thus, *ftn-1* regulation in *C. elegans* can serve as a model for understanding the mechanisms of iron homeostasis in humans, and of human disease.

In this study, we explore the biology of iron homeostasis in *C. elegans* by investigating further the regulation of *ftn-1*. We show that *ftn-1* is transcriptionally regulated by IIS/DAF-16, and then perform a genetic screen using RNA mediated interference (RNAi) to identify factors influencing expression of a *Pftn-1::gfp* reporter. We identify several transcription factors known to act with IIS to regulate lifespan as factors that also regulate *ftn-1* expression. We also reveal a major role for the hypoxia signaling pathway in *ftn-1* regulation and iron homeostasis.

## Results

### *ftn-1* expression is regulated by insulin/IGF-1 signaling

**Figure 1:**
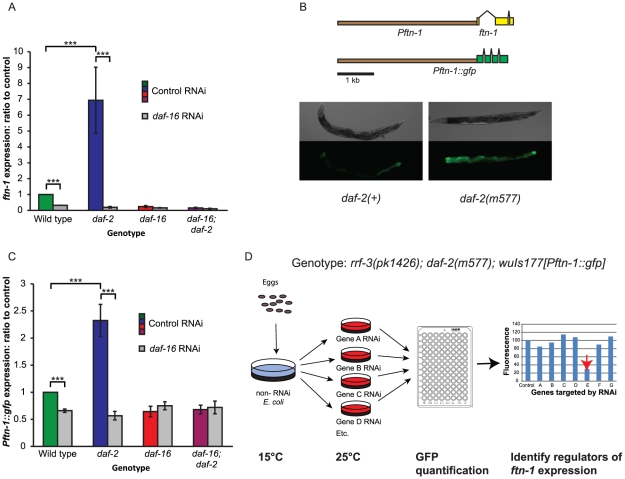
Regulation of the ferritin gene *ftn-1* by insulin/IGF-1 signaling. (A) Effect of loss of *daf-2* and *daf-16* function on *ftn-1* mRNA levels. Animals were grown at 15°C until the L4 stage of development, then kept at 25°C for two days prior to RNA extraction. (B) Construction of *Pftn-1::gfp* transgenic *C. elegans*. Approximately 3.8 kb of upstream sequence was fused to the GFP coding sequence. Epifluorescence images of nematodes bearing *wuIs177 [Pftn-1::gfp]* in *daf-2(+)* or *daf-2(m577)* backgrounds. Animals were grown at 15°C until the L4 stage and then kept at 25°C for two days before microscopy. The same exposure time was used for both images. (C) Effect of loss of *daf-2* and *daf-16* function on *Pftn-1::gfp* expression (c.f. Figure 1A). Animals were grown at 15°C until the L4 stage of development and then transferred to RNAi plates. They were then kept at 25°C for two days before GFP fluorescence measurements. (D) Diagrammatic depiction of RNAi screening protocol. Eggs were isolated by alkaline hypochlorite treatment and synchronized populations were left to develop at 15°C until the L4 stage of development. L4 animals were transferred to RNAi plates and left at 25°C for two days. Quantification of GFP expression was carried out by picking 40 animals into microtitre plates and measuring fluorescence using a platereader with a GFP filter set. Statistical significance was calculated by ANOVA in all cases. ***: p<0.001.

To ascertain whether *ftn-1* expression might be regulated by insulin/IGF-1 signaling (IIS) and *daf-16*, we examined published microarray-derived mRNA profiles comparing *daf-2* and *daf-16; daf-2* mutants [26], [27]. These implied that *ftn-1* mRNA levels are greatly elevated (47-fold increase) in *daf-2* compared to *daf-16; daf-2* animals. This we were able to confirm using qRT-PCR (Figure 1A). The increase in *ftn-1* mRNA levels in *daf-2* mutants was fully *daf-16* dependent. Loss of *daf-16* also decreased *ftn-1* mRNA levels in *daf-2(+)* animals.

We then created a transgenic *C. elegans* line bearing a *Pftn-1::gfp* transcriptional reporter containing 3.8 kb of sequence upstream of the *ftn-1* start codon. This was generated by microinjection of transgene DNA, and the resulting extrachromosomal transgene arrays were then chromosomally integrated. The *Pftn-1::gfp* transgene showed strong expression throughout the intestine, consistent with previous reports [20]. Effects of *daf-2* and *daf-16* upon *Pftn-1::gfp* expression paralleled those seen in *ftn-1* mRNA levels (Figure 1B, 1C). This confirms that *ftn-1* is regulated by IIS, and shows that this regulation occurs principally in the intestine.

### RNAi screen identifies more regulators of *ftn-1* expression

We then used the *Pftn-1::gfp* reporter as the basis of an RNAi screen to investigate the mechanisms by which *ftn-1* is regulated (Figure 1D). The initial aim of this screen was to identify pathways that work coordinately with IIS, and regulatory factors that act downstream of DAF-16. Expression of the integrated GFP (green fluorescent protein) reporter was intensified by mutation of *daf-2* and sensitivity to RNAi was increased by introducing the *rrf-3(pk1426)* mutation. The resulting strain, of genotype *rrf-3(pk1426); daf-2(m577ts); wuIs177 [Pftn-1::gfp]*, was raised at 15°C until the L4 stage, then transferred to RNAi plates and incubated at 25°C (non-permissive temperature for *daf-2(m577)*). GFP fluorescence levels were measured in a plate-reader two days later.

**Figure 2:**
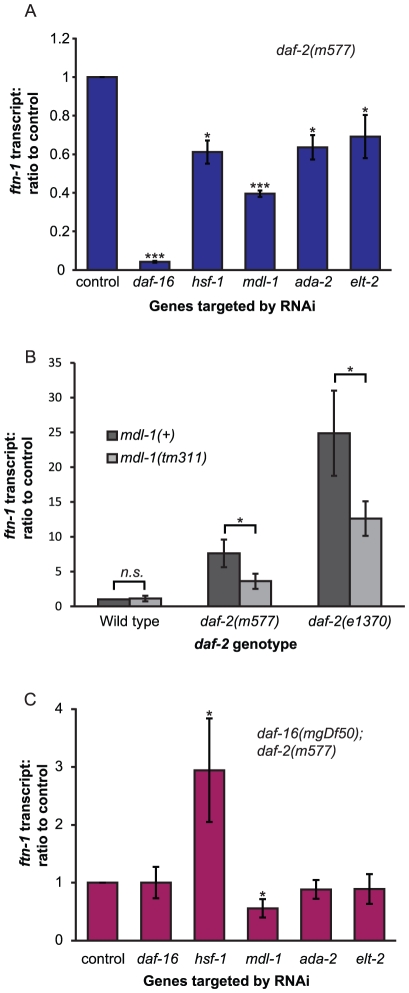
Identification of genes activating *ftn-1* expression. (A) Effect of RNAi of several transcription factors on *ftn-1* transcript levels in *daf-2(m577)* mutants. (B) Effect of loss of *mdl-1* on *ftn-1* expression in both *daf-2(m577)* and *daf-2(e1370)* mutants. Protocol as described for (A) above. (C) Effect of RNAi on *ftn-1* transcript levels in *daf-16(mgDf50); daf-2(m577)* mutants. For all trials, samples were collected on day 2 of adulthood. Statistical significance calculated by ANOVA. *n.s*:. non-significant, *: p<0.05, ***: p<0.001.

Given our interest in mechanisms of gene regulation, the RNAi screen was restricted to 812 genes encoding predicted transcription factors or other proteins associated with gene regulation [28]. RNAi of a number of these genes led to altered *Pftn-1::gfp* expression. In an initial screen, RNAi of 30 genes reduced *Pftn-1::gfp* expression by ≥20% (Table S1) and we investigated these more thoroughly in several genetic backgrounds. For 10 of these genes, not including *daf-16*, RNAi consistently and robustly reduced *Pftn-1::gfp* expression in multiple trials (data not shown). We then verified the effect of RNAi on levels of mRNA from the endogenous *ftn-1* gene. This identified four genes where RNAi robustly reduced *ftn-1* mRNA levels: *hsf-1*, *mdl-1*, *ada-2* and *elt-2* (Figure 2A, Table S1).

The heat-shock factor *hsf-1* was previously shown to mediate effects of IIS on gene expression [29]. The screen also confirmed that the GATA transcription factor *elt-2* plays a role in *ftn-1* regulation. This is consistent with the role of *elt-2* as an activator of intestinal gene expression [30]; moreover, *elt-2* is the only previously described transcriptional activator of *ftn-1* expression [20]. Thus, identification of *hsf-1* and *elt-2* in this unbiased screen is evidence of the efficacy of the screen. *ada-2* encodes a homolog of the Ada2 subunit of various histone acetyl transferase (HAT) complexes that activate gene expression by modifying chromatin via histone acetylation [31]. It is possible that, *ada-2* influences *ftn-1* expression via effects on chromatin status.

More notable is the identification of the MAD-like transcription factor *mdl-1* as an activator of *ftn-1* expression. *mdl-1* plays a role in the protective effects of reduced IIS against a tumorous germline phenotype [32] and is upregulated in IIS mutants [10], [26], [32]. We confirmed that the null mutation *mdl-1(tm311)* reduces *ftn-1* mRNA levels in *daf-2* mutants (Figure 2B).

To explore whether these four factors might be acting downstream of DAF-16, we tested whether RNAi reduces *ftn-1* expression in a *daf-16; daf-2* double mutant. The results imply that only MDL-1 does not require DAF-16 to activate *ftn-1* expression. This suggests that *mdl-1* acts downstream of *daf-16*, or possibly in parallel to IIS, to regulate *ftn-1* expression (Figure 2C). Given that *mdl-1* is a direct transcriptional target of DAF-16 [33], the former seems more likely.

Unexpectedly, RNAi of *hsf-1* markedly increased *ftn-1* expression in a *daf-16; daf-2* background (Figure 2C). The effects of *hsf-1* RNAi (Figure 2A) imply that HSF-1 and DAF-16 act together to activate *ftn-1* expression, as previously shown for other genes [29]. That loss of *hsf-1* in *daf-16; daf-2* mutants increases expression of *ftn-1* could imply a repressive role of HSF-1 in the absence of DAF-16. Alternatively, this increase might merely reflect a stressed state in the worms, caused by loss of both *hsf-1* and *daf-16* at 25°C (see Discussion).

Since *ftn-1* is known to be responsive to iron levels, we also tested whether DAF-16, HSF-1 or MDL-1 are required for iron-dependent regulation of *ftn-1*. *daf-16*, *hsf-1* and *mdl-1* mutants were treated with iron (25 mM ferric ammonium citrate, FAC) and *ftn-1* transcript levels measured by qRT-PCR. Iron-induced up-regulation of *ftn-1* was unchanged in each case (Figure S1A), i.e. these three factors do not mediate effects of iron on *ftn-1* expression.

### *hif-1* and *daf-2* act additively to repress *ftn-1* expression

RNAi of 28 genes further increased expression of the *Pftn-1:gfp* reporter (Table S2), already induced by *daf-2(m577)*. Of note was the large increase in expression upon RNAi of *unc-62*, a transcription factor with a conserved role in ferritin regulation and, for its human ortholog, a possible role in the iron-related disorder ‘restless leg syndrome’ [25].

**Figure 3:**
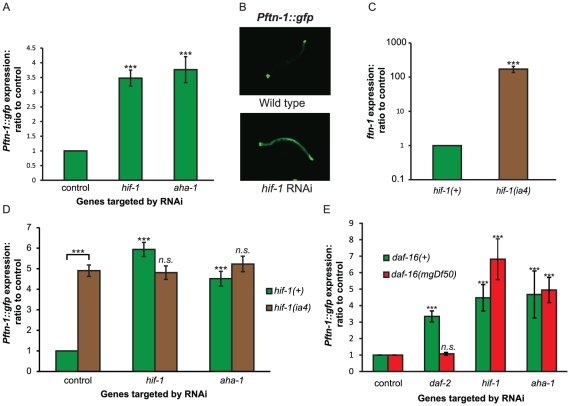
HIF signaling regulates *ftn-1* expression. (A) Effect of RNAi of *hif-1* and *aha-1* on the expression of *Pftn-1::gfp*. This result was obtained from animals carrying *wuIs177 [Pftn-1::gfp]*. Animals were grown at 20°C, transferred to RNAi at the L4 stage and GFP fluorescence quantified two days later. (B) Epifluorescence image of *Pftn-1::gfp* under control conditions (L4440) and *hif-1* RNAi. Animals were grown at 20°C and photographed on day 2 of adulthood. (C) Effect of *hif-1(ia4)* on *ftn-1* transcript levels. Animals were grown at 20°C and samples were collected on day 1 of adulthood. (D) Effect of *hif-1* and *aha-1* RNAi on *ftn-1* expression in wild-type and *hif-1(ia4)* animals. (E) Effect of *hif-1*, *aha-1* and *daf-16* RNAi on *Pftn-1::gfp* expression in wild type and *daf-16* mutants. These measurements were carried out on L4 animals kept at 25°C. In order to quantify GFP expression in L4 animals, 60 rather than 40 animals were transferred to each well of the microtitre plates. Statistical significance calculated by ANOVA. *n.s*:. non-significant, ***: p<0.001.

Also among the repressors of *Pftn-1::gfp* expression identified were *hif-1*, encoding the hypoxia-inducible factor, and *aha-1*, its binding partner (HIFβ, also called ARNT). RNAi of either gene strongly increased *Pftn-1::gfp* expression, in the original *daf-2(m577); Pftn-1::gfp* strain (Table S2) but also in two separate integrants of the *Pftn-1::gfp* reporter in a *daf-2(+)* background (Figure 3A, 3B and data not shown).

We verified this activity of *hif-1* by using the loss of function mutation *hif-1(ia4)*, which proved to greatly increase *ftn-1* mRNA levels (Figure 3C) and *Pftn-1::gfp* expression (Figure 3D). In a *hif-1(ia4)* mutant background, RNAi of *aha-1* did not further increase *Pftn-1::gfp* expression (Figure 3D), indicating that *hif-1* and *aha-1* act together to repress *ftn-1* expression.

The finding that *hif-1* RNAi increases *Pftn-1::gfp* expression in a *daf-2* mutant background suggests that *hif-1* influences *ftn-1* expression independently of IIS. Consistent with this, *hif-1* or *aha-1* RNAi increased *Pftn-1::gfp* expression in the absence of *daf-16* (Figure 3E). Results were similar at both 25°C and 20°C and at L4 and adult stages (Figure 3E and data not shown). In addition, RNAi of *hif-1* increased *ftn-1* transcript levels in *daf-16* mutants, and also in *hsf-1* and *mdl-1* mutants (Figure S1B), indicating that none of these factors act downstream of HIF-1 to regulate *ftn-1* expression.

### Stabilization of HIF-1 reduces *Pftn-1::gfp* expression

**Figure 4:**
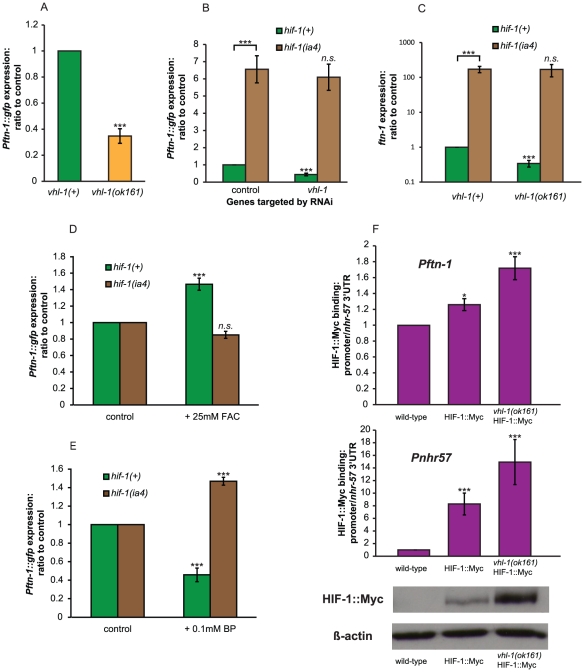
Evidence that the HIF pathway acts as an iron sensor. (A) Effect *vhl-1(ok161)* on *Pftn-1::gfp* expression. Experiment was carried out at 20°C. (B) Effect of RNAi of *vhl-1* on *Pftn-1::gfp* expression in wild-type and *hif-1(ia4)* animals. Nematodes were maintained on RNAi plates for two generations at 25°C and GFP fluorescence quantified at the L4 stage. (C) *ftn-1* transcript levels in wild type, *vhl-1(ok161)*, *hif-1(ia4)* and *hif-1(ia4); vhl-1(ok161)* double mutants. Cultures were grown at 20°C and samples were collected at day 1 of adulthood. (D) Effect of addition of iron (25 mM FAC) on expression of *Pftn-1::gfp* in wild type and *hif-1(ia4)* mutants. (E) Effect of addition of 0.1 mM bipyridyl (BP) on expression of *Pftn-1::gfp* in wild type and *hif-1(ia4)* mutants. (F) Chromatin immunoprecipitation (ChIP) was carried out using N2 (wild type), ZG429 (*hif-1::Myc*) and GA654 (*hif-1::Myc vhl-1(ok161)*. Binding was assessed by qRT-PCR of ChIP samples using primers against the promoters of *ftn-1* and the known HIF-1 target gene *nhr-57*. Values obtained were normalized to using qRT-PCR with primers against the 3′UTR of *nhr-57*, to which HIF-1::Myc is not thought to bind. HIF-1::Myc protein levels were quantified by Western blot using the same antibody aliquot as used for the ChIP experiment. Statistical significance calculated by ANOVA. *: p<0.05, ***: p<0.001.

If HIF-1 is a repressor of *ftn-1* expression, then elevation of HIF-1 levels should decrease expression of *Pftn-1::gfp*. Loss of *vhl-1* (von Hippel-Lindau factor) leads to increased HIF-1 protein levels in *C. elegans* [34]. As expected, the deletion mutation *vhl-1(ok161)* markedly decreased expression of *Pftn-1::gfp* (Figure 4A). Moreover, RNAi of *vhl-1* reduced *Pftn-1::gfp* expression in *hif-1*(+) but not *hif-1(ia4)* animals (Figure 4B), and genetic deletion of *vhl-1* led to a reduction in *ftn-1* transcript levels that is also completely dependent on *hif-1* (Figure 4C). These results imply that HIF-1 acts downstream of VHL-1 as a repressor of *ftn-1* expression.

### The induction of *Pftn-1::gfp* expression by iron requires hif-1

The prolyl hydroxylase EGL-9 hydroxylates the P621 residue of HIF-1, which causes VHL-1 to bind to HIF-1, leading to proteasomal degradation [34]. This hydroxylation reaction requires iron as a cofactor, suggesting that regulation of *ftn-1* expression by iron might involve the HIF-1 pathway. We therefore tested whether the effects of iron on *ftn-1* expression are *hif-1* dependent.

As expected given previous findings [23], both expression of *Pftn-1::gfp* and *ftn-1* mRNA levels were increased upon supplementation with iron (ferric ammonium citrate, FAC) and decreased upon treatment with the iron chelator 2′-2 bipyridil (BP) (Figure 4D, 4E and Figure S2A and S2B). This is consistent with the previous observation that BP treatment greatly increases HIF-1 protein levels in *C. elegans* [34], since increased HIF levels would be expected to further repress *ftn-1* expression. By contrast, in *hif-1(ia4)* mutants, addition of iron did not increase either *Pftn-1::gfp* expression or *ftn-1* mRNA levels. This implies that *hif-1* mediates the induction of *ftn-1* expression by iron. Iron chelation did not decrease *ftn-1::gfp* and *ftn-1* expression in *hif-1(ia4)*, but instead increased it. The cause of this induction remains unexplained. One possibility is that BP treatment leads to cellular stress and induction of other stress response regulators (e.g. DAF-16), which can activate *ftn-1* expression in the absence of the repressive effects of HIF-1 (see Discussion).

### HIF-1 binds to the *ftn-1* promoter

In order to investigate whether HIF-1 represses *ftn-1* expression by directly binding to the *ftn-1* promoter, we carried out a chromatin immunoprecipitation (ChIP) assay using *C. elegans* expressing Myc-tagged HIF-1 [35] and an anti-Myc antibody. We used three lines: wild type (N2), ZG429 [*hif-1::myc*] and GA654 [*hif-1::myc vhl-1(ok161)*]. Given that *vhl-1* mutants have elevated HIF-1 levels and reduced *ftn-1* mRNA levels (Figure 4C), greater levels of HIF-1::Myc binding to the *ftn-1* promoter should be detectable in *vhl-1* mutants, if the interaction is in fact direct.

We first checked that our ChIP protocol allowed us to measure binding by HIF-1::Myc by testing binding to the promoter of a known HIF-1 target gene, *nhr-57*. We designed one set of primers to amplify the region of the promoter containing two putative hypoxia response elements (HREs) and another set of primers targeting an area within the 3′ UTR of this gene. Quantity of qRT-PCR amplified promoter DNA was then compared to the 3′ UTR quantity as a test of enrichment of the promoter in our ChIP DNA pools. This amplification from the 3′ UTR (to which HIF-1 is not expected to bind) allowed us to control for input quantity. We saw a large (8.3-fold) enrichment of the *nhr-57* promoter sequence in the HIF-1::Myc lines and an even greater (14.9-fold) enrichment when HIF-1::Myc was stabilized by deletion of *vhl-1* (Figure 4F). Relative amounts of HIF-1::Myc were monitored by Western blotting of the same ChIP samples using the same aliquot of anti-Myc antibody used for ChIP, and we were able to confirm that *vhl-1(ok161)* increases HIF-1::Myc protein levels (Figure 4F).

We then measured binding to the *ftn-1* promoter through qRT-PCR against the promoter sequence of *ftn-1*. For this, we used a primer pair specific to the IDE sequence. These results were again normalized against the same *nhr-57* 3′UTR in order to correct for differences in input quantity. While weaker than binding to *Pnhr-57*, enrichment of the *Pftn-1* sequence in HIF-1::Myc and stabilized HIF-1::Myc lines was statistically significantly different to that seen in wild-type controls (Figure 4F). This is evidence that HIF-1 represses *ftn-1* expression through direct binding to its promoter.

### Iron-dependent regulation of *ftn-1* is partially *vhl-1*–dependent

The repression of *ftn-1* expression by HIF-1 and the requirement for iron in the degradation of HIF-1 by the proteasome suggests a possible mechanism for the iron-dependent regulation of *ftn-1* in which changes in iron levels alter the level of HIF-1 protein, which in turn alter *ftn-1* expression. Since the iron-dependent degradation of HIF-1 occurs via the action of VHL-1, HIF-1 protein levels in *C. elegans* are not sensitive to iron levels when VHL-1 is absent [36].

**Figure 5:**
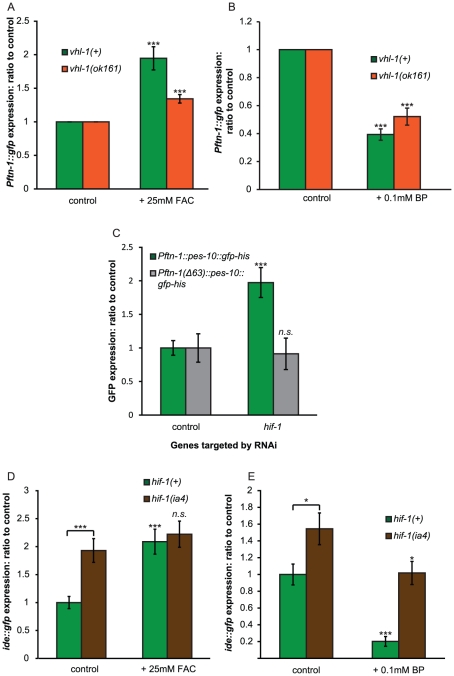
HIF-1 represses expression via the iron-dependent element (IDE). (A) Expression of *Pftn-1::gfp* in wild-type and *vhl-1(ok161)* animals with or without addition of iron (25 mM FAC). GFP fluorescence was quantified using a plate reader after 18 h of iron treatment. At least ten biological replicates were quantified. Asterisks denote statistically significant difference when compared to non-iron treated controls. (B) Expression of *Pftn-1::gfp* in wild-type and *vhl-1(ok161)* animals with or without iron chelation (0.1 mM bipyridyl, BP). GFP fluorescence was quantified using a plate reader after 18 h of iron chelation. BP-treated animals were compared to ethanol control treated ones. (C) Effect of *hif-1* RNAi on the *Pftn-1::gfp* transgene with or without the IDE regulatory element. Fluorescence was measured in L4 animals grown at 25°C through pixel density quantification of epifluorescence microscopy images. *hif-1* RNAi was administered for one generation. Strains used: XA6900 and XA6902. (D) *ide::gfp* expression in *hif-1(+)* and *hif-1(ia4)* animals with or without the addition of iron. Fluorescence measured as in (C). FAC treatment was administered from egg to L4 stage of development. (E) *ide::gfp* expression in *hif-1(+)* and *hif-1(ia4)* animals with or without the addition of iron chelator. Due to toxic effects of BP treatment during development, BP treatment was administered for 18 h during adulthood. Quantification was carried out on the second day of adulthood using pixel density quantification. *n.s*:. non-significant, *: p<0.05, ***: p<0.001.

We found that loss of *vhl-1* largely abrogated the induction of *Pftn-1::gfp* expression by iron supplementation, though there was still a significant induction of lesser magnitude (Figure 5A). Reduction of *Pftn-1::gfp* expression by iron chelation was not affected by loss of *vhl-1* (Figure 5B). Taken together, this suggests that regulation of *ftn-1* by iron may occur partially, but not exclusively, through changes in HIF-1 protein levels regulated by iron-dependent degradation.

### Evidence that *hif-1* mediates iron-dependent regulation via the iron-dependent element

The induction of *ftn-1* levels by iron requires a 63 bp element (the iron-dependent element, or IDE) in the gene's promoter [20]. We wondered whether the *hif-1* pathway might mediate the effects of iron on IDE-mediated gene expression. A reporter strain carrying a *ftn-1* promoter lacking the IDE is insensitive to changes in iron levels [20]. Using these same reporters we found that absence of the IDE abolished *hif-1* RNAi-induced induction of expression (Figure 5C).

Another reporter construct with just the IDE sequence fused to a minimal promoter and driving GFP expression was previously shown to be responsive to iron [20]. We found that loss of *hif-1* increased *ide::gfp* expression, demonstrating that *hif-1* does promote gene expression from the IDE (Figure 5D). Moreover, addition of iron did not induce *ide::gfp* expression in *hif-1* mutants (Figure 5D). However, in *hif-1* mutants treatment with the iron chelator BP still reduced *ide::gfp* expression (Figure 5E). This possibly reflects an effect of BP on *ftn-1* that is independent of its effects on iron levels, or the existence of a second iron-dependent factor. These results show that the IDE is subject to regulation by HIF-1 and suggest that HIF-1 mediates the effects of iron on IDE-mediated gene expression.

### Loss of *egl-9* increases *ftn-1* expression

**Figure 6:**
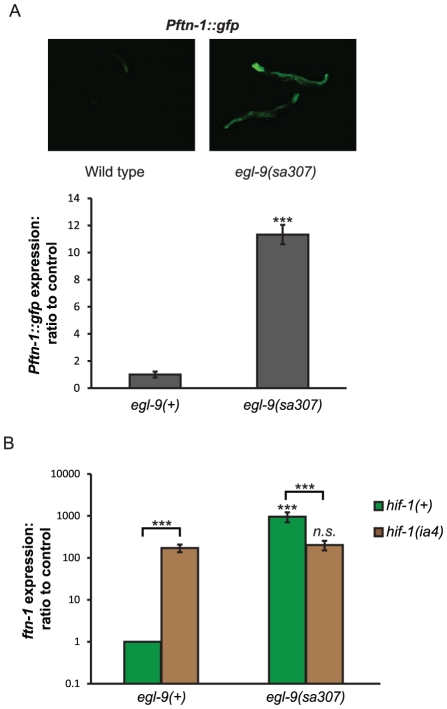
Regulation of *ftn-1* expression by EGL-9. (A) Effect of *egl-9(sa307)* deletion mutaion on *Pftn-1::gfp* expression. Epifluorescence microscopy and plate reader quantification of GFP fluorescence was carried out on day 2 of adulthood. (B) Effect of *egl-9* deletion on *ftn-1* transcript levels in *hif-1(+)* and *hif-1(ia4)* animals. Samples were collected at day 1 of adulthood. Statistical significance calculated by ANOVA. *n.s*:. non-significant, ***: p<0.001.

As previously described, loss of *vhl-1* decreases expression of *Pftn-1::gfp* (Figure 4A). This is expected given that HIF-1 represses *ftn-1* expression and that loss of *vhl-1* increases HIF-1 levels [34]. The prolyl hydroxylase EGL-9 targets HIF-1 for proteasomal degradation, and loss of *egl-9* causes a similarly large increase in HIF-1 protein levels as loss of *vhl-1* [36]. We therefore expected that loss of *egl-9*, like that of *vhl-1*, would reduce *Pftn-1::gfp* expression. In fact, deletion of *egl-9* caused an 11-fold increase in *Pftn-1::gfp* expression (Figure 6A) and a ∼950-fold increase in *ftn-1* mRNA levels (Figure 6B). Animals with a different allele, *egl-9(n586)*, also showed increased *ftn-1* mRNA levels (Figure S3A). Visible *Pftn-1::gfp* expression remained restricted to the intestine in wild type, *vhl-1* mutants and *egl-9* mutants.

*vhl-1*-independent effects of EGL-9 on HIF-1 target gene expression have been observed previously [36]. Our findings suggest that in the case of *ftn-1* regulation, *egl-9* can act independently of, and antagonistically to, *vhl-1*. As expected, loss of *egl-9* induced *ftn-1* expression even in the absence of *vhl-1* (Figure S3B and S3C). However, *egl-9* RNAi did not increase *ftn-1* transcript or *Pftn1::gfp* expression in the absence of *hif-1* (Figure 6B and Figure S3D). This implies that the inhibition of *ftn-1* expression by EGL-9 also requires *hif-1*.

Thus, *egl-9* and *vhl-1* inhibit and activate expression of *ftn-1*, respectively, and both activities require *hif-1*. One possibility is that EGL-9 inhibits *ftn-1* expression by stimulating HIF-1 activity via an as yet unidentified pathway.

## Discussion

In this study, we have investigated the regulation of the inducible *C. elegans* ferritin gene *ftn-1*, a key determinant of iron homeostasis. We reveal that expression of this gene is coordinately regulated by insulin/IGF-1 and HIF signaling, pathways previously known to interact in the regulation of stress resistance and lifespan. Our findings imply that the HIF pathway is required for gene regulation in response to iron levels in *C. elegans*, and that IIS controls iron homeostasis, potentially increasing free iron availability to support growth.

### Insulin/IGF-1 signaling (IIS) regulates growth and iron homeostasis

IIS and DAF-16 play a pivotal role in the organismal decision between growth and diapause. Under growth-promoting conditions, DAF-16 is inactivated through cytoplasmic retention, which facilitates reproductive growth [8], [37]. In the absence of sufficient food or given exposure to certain forms of stress, DAF-16 enters the nucleus and transcriptionally specifies a survival program. This entails delayed reproduction, enhanced stress resistance and increased lifespan. Modulation of DAF-16 activity is therefore crucial for ensuring an optimal response to the worm's environment; with growth and reproduction under conditions that are propitious to growth, and developmental arrest, stress protection and increased longevity under conditions that are not.

Regulation of *ftn-1* by DAF-16 suggests the existence of a trade-off between growth and stress resistance involving iron homeostasis. A role for ferritin in regulating growth via its effects on iron homeostasis has been described previously in mammalian cells [38]. This study found that Myc, a bHLH transcription factor with a major role in promoting cellular proliferation, can repress H-ferritin expression. Overexpression of ferritin in cells carrying activated Myc led to a decrease in *in vitro* clonogenicity, and this effect could be rescued by addition of iron, suggesting that Myc–mediated repression of ferritin expression favors growth by increasing iron availability. The study identified DNA synthesis as a possible mechanism for iron-dependent control of cellular proliferation by c-Myc, as DNA synthesis is increased by c-Myc in a manner dependent on ferritin repression and the associated increases in iron availability. This finding is consistent with the requirement for iron in the activity of ribonucleotide reductase, the rate-limiting enzyme in DNA synthesis. Similar mechanisms may be at play in the regulation of ferritin expression by IIS. When conditions favor growth, and IIS is increased, reduced *ftn-1* expression is expected to increase iron availability, thus fulfilling a key requirement for growth.

While free iron is required for growth, it can also cause harm by catalyzing the Fenton reaction, which increases levels of ROS and molecular damage. When conditions are not suitable for growth, IIS is reduced, and increased *ftn-1* expression is expected to lower levels of free iron and of ROS, thereby protecting against stress. Consistent with this, induced over-expression of *ftn-1* causes resistance to oxidative stress (S. Valentini and D. Gems, unpublished results). Thus, upregulation of *ftn-1* likely contributes to the broader increase in cytoprotection seen when IIS is reduced.

Reduced IIS also increases levels of autophagy in *C. elegans* [39], [40] and autophagy releases iron from ferruginous materials, such as mitochondrial metalloproteins [41]. This predicts that reduced IIS will increase free iron levels, and concomitant elevation of *ftn-1* expression could ensure that iron released by autophagy does not cause molecular damage.

### Transcriptional activators of *ftn-1* expression

Using an RNAi screen we identified new regulators of *ftn-1*, including *hsf-1* and *mdl-1*. It was previously shown that in *daf-2* mutants the heat shock factor HSF-1 acts in concert with DAF-16 to promote expression of small heat shock proteins and other molecular chaperones, which contribute to longevity [29]. We find that *hsf-1* is also involved in the induction of *ftn-1* in *daf-2* mutants, since loss of *hsf-1* reduced *ftn-1* expression in *daf-2* but not *daf-16; daf-2* mutants.

The MAD-like transcription factor *mdl-1* is also regulated by IIS. Microarray and qRT-PCR studies showed it to be up-regulated in *daf-2* mutants [10], [26], [32]. *mdl-1* also contributes to the resistance of *daf-2* mutants to germline tumor formation in the *gld-1* tumor model, and to *daf-2* mutant longevity [32]. That MDL-1 activates *ftn-1* expression is consistent with the role of mammalian MAD as an inhibitor of Myc, which represses ferritin expression (see above); however, *C. elegans* does not possess any clear ortholog of Myc [42], [43].

A study of DAF-16 binding sites did not provide evidence that *ftn-1* is a direct regulatory target of DAF-16 [33], but suggested that *mdl-1* might be. Given that *ftn-1* may be a direct target of MDL-1 [44], [45], one possibility is that activation of *mdl-1* expression by DAF-16 leads to increased *ftn-1* expression. This hypothesis predicts that abrogation of *mdl-1* expression should decrease *ftn-1* expression more in *daf-2* than in *daf-16; daf-2* animals, but this is not the case (Figure 2A, 2C). This could imply that *mdl-1* regulates *ftn-1* independently of *daf-16*, at least in part.

### *ftn-1* is negatively regulated by *hif-1* and *aha-1*

We discovered that loss of *hif-1* or its binding partner *aha-1* greatly increased *ftn-1* expression in *daf-2* mutants. This implicated hypoxia signaling in the regulation of *ftn-1*.

The HIF transcription factor is composed of an α and a β subunit, encoded by the genes *hif-1* and *aha-1* in *C. elegans*. HIF regulates the transcriptional response to hypoxia in both worms and vertebrates and, as expected, worms lacking *hif-1* are hypersensitive to hypoxia [46]. Levels of HIFβ protein are relatively stable, whereas HIFα is constantly being degraded by the proteasome under normal, non-hypoxic conditions. In both worms and higher organisms, this occurs because the HIFα/HIF-1 protein is hydroxylated at conserved proline residues by prolyl hydroxylase (PHD), encoded by the *egl-9* gene in worms. After hydroxylation by PHD/EGL-9, the von Hippel-Lindau protein VHL-1 binds to HIFα, which targets it for degradation [34], [47].

PHDs require oxygen, iron and 2-oxoglutarate for the hydroxylation reaction. When cells are kept under hypoxic conditions or when an iron chelator is added, the proline residue in HIFα is not hydroxylated and the HIFα protein accumulates [48]. That loss of *hif-1* has such dramatic effects on gene expression under normoxic conditions demonstrates that HIF-1 affects gene regulation even at the very low levels of HIF-1 found when it is being hydroxylated and degraded. Similarly, it was previously observed that loss of *hif-1* can increase *C. elegans* lifespan under normoxic conditions [49]. Consistent with this, we find statistically significant levels of binding of the non-stabilized HIF-1::Myc protein to both *ftn-1* and *nhr-57* promoters (Figure 4F).

Since iron is a required cofactor for hydroxylation of HIF by PHD, levels of iron affect those of HIF. For example, in *C. elegans*, depletion of iron using the iron chelator 2-2′ bipyridyl stabilizes HIF-1 [34], and feeding mice a low-iron diet leads to increased HIFα levels [50]. This increase in HIF-1 levels is not without consequence: chelation of iron has also been shown to increase expression of the *C. elegans* HIF-1 target gene *nhr-57*, indicating that the stabilization of HIF upon loss of iron leads to HIF-1-dependent changes in gene expression [51]. In vertebrates, HIF activates expression of genes involved in regulating iron homeostasis, including heme oxygenase [52], the transferrin receptor [53], [54], ceruloplasmin [55], DMT1 [56] and possibly ferroportin [57]. Loss of HIF-2α in mice causes decreased iron levels in the plasma and livers of mice [56]. It has therefore been suggested that HIF can act as an iron sensor: low iron levels lead to HIF stabilization, which leads to changes in gene expression that increase iron levels [57]. The results presented here support this hypothesis.

### Iron-dependent regulation of *ftn-1* via *hif-1*

The repression of ferritin expression by *hif-1/aha-1* is consistent with a role of HIF in increasing iron availability. By this view, lower ferritin expression upon HIF activation would reduce iron storage capacity, thereby increasing iron availability. We therefore investigated whether HIF mediates iron-dependent regulation of *ftn-1*, and this proved to be the case: *ftn-1* regulation by iron is blocked in *hif-1* mutants. In wild-type animals iron supplementation increases *ftn-1* expression while iron depletion decreases it. By contrast, in *hif-1* mutants iron supplementation does not increase *ftn-1* expression.

Treatment of *hif-1* mutants with the iron chelator 2-2′ bipyridyl (BP) caused a large increase, rather than decrease, of *ftn-1* expression. This was unexpected, but we noticed that BP treated worms were somewhat sickly in appearance. One possibility is that toxicity of BP in *hif-1* mutants triggers other stress response mediators (e.g. DAF-16) that activate *ftn-1* expression. This is consistent with our observation that stressful conditions tend to induce expression of this reporter. Similar to treatment with BP, RNAi of *hsf-1* in *daf-16(mgDf50); daf-2(m577)* animals raised at 25°C also caused the worms to have a sickly appearance and induced *Pftn-1::gfp* expression (Figure 2C). Moreover, we observed that starved animals also show elevated *Pftn-1::gfp* expression (data not shown).

The requirement for *hif-1* in the iron-dependent regulation of *ftn-1* suggests that this regulation may occur via iron-dependent degradation of HIF-1. However, our data implies that this is not the whole story. Mutants of *vhl-1* have constitutively stabilized HIF-1 and its levels cannot therefore respond to changes in iron (or oxygen) levels [34], [36]. While the increase in *Pftn-1::gfp* expression upon treatment with iron was greatly reduced in *vhl-1* mutants, *Pftn-1::gfp* expression was still elevated compared to the control treatment. This implies that iron-dependent degradation of HIF-1 is not the sole mechanism by which *ftn-1* is regulated in response to iron levels.

The control of *ftn-1* expression by iron was previously shown to be mediated by the 63 bp iron-dependent element (IDE) in the *ftn-1* gene promoter [20]. This implied the presence of an unknown iron-responsive transcriptional activator exerting effects upon the IDE. Our findings strongly suggest that this factor is HIF. We found that loss of *hif-1* increases *ide::gfp* expression. Moreover, in the absence of *hif-1*, iron supplementation failed to induce *ide::gfp* expression. Furthermore, Romney et al. (2008) identified three conserved elements (called DR elements), with the consensus sequence: CACGTA(C/G)(C/A/G) in the IDE to which they attribute the responsiveness of *ftn-1* expression to iron levels. This DR sequence has homology to the E-box motif, which led Romney et al. to suggest that the iron-sensory pathway includes a basic helix-loop-helix (bHLH) transcription factor. Both HIF-1 and AHA-1 belong to this family of proteins. In fact, the conserved DR sequence described by Romney et al. contains the putative *C. elegans* hypoxia response element (HRE) [58] (in reverse orientation). Moreover, using ChIP, we found that epitope-tagged HIF-1 bound to the region of the promoter containing the IDE. Taken together, these results support the view that HIF-1 acts as an iron sensor in *C. elegans*, suppressing *ftn-1* expression by binding to the IDE, although the mechanism by which iron levels are detected has not yet been identified.

Our discovery of the role of HIF in iron homeostasis in *C. elegans* has notable implications in terms of the evolution of HIF as an iron sensor. The effects of iron on HIF levels in higher organisms has been viewed in the context of HIF's role in stimulating erythropoiesis. Since erythropoiesis requires large quantities of iron, it was proposed that the purpose of the HIF-mediated induction of genes involved in increasing iron availability is to supply iron for erythropoiesis [57]. That HIF regulates iron homeostasis in nematodes implies that the evolution of this function predates the emergence of a circulatory system. The sensitivity of hypoxia signaling to oxygen, iron and ROS, which interact and produce oxidative damage to the cell, further suggests that HIF may have an ancestral role in fine-tuning the response to different levels of these potentially toxic substances.

### Antagonistic regulation of *ftn-1* by *vhl-1* and *egl-9*

Against expectation, loss of *egl-9* increased expression of *Pftn-1::gfp*, rather than decreasing it. This does not merely indicate that EGL-9 regulates targets other than HIF-1, since the induction is *hif-1* dependent. Given that loss of *egl-9* or *vhl-1* cause similar increases in HIF-1 protein levels [36], this finding suggests that increased HIF protein levels can be associated with both increased and decreased *ftn-1* expression.

That both decreased expression upon *vhl-1* deletion and increased expression upon *egl-9* deletion require *hif-1* is difficult to reconcile. However, *vhl-1* independent effects of EGL-9 on HIF-1 target gene expression have been observed previously [36]. HIF-1 target genes are often more highly induced by loss of *egl-9* than of *vhl-1*, despite identical levels of HIF-1 stabilization in each case [36]. This implies that regulation of HIF-1 target gene expression by EGL-9 occurs via both VHL-1-dependent and independent mechanisms. Additionally, mutations in *egl-9* protect worms against infection by *Pseudomonas aeruginosa* and this effect is dependent on HIF-1. However, stabilization of HIF-1 by other means is insufficient to achieve this effect, again showing that EGL-9 can act via mechanisms other than HIF-1 stabilization [59].

**Figure 7:**
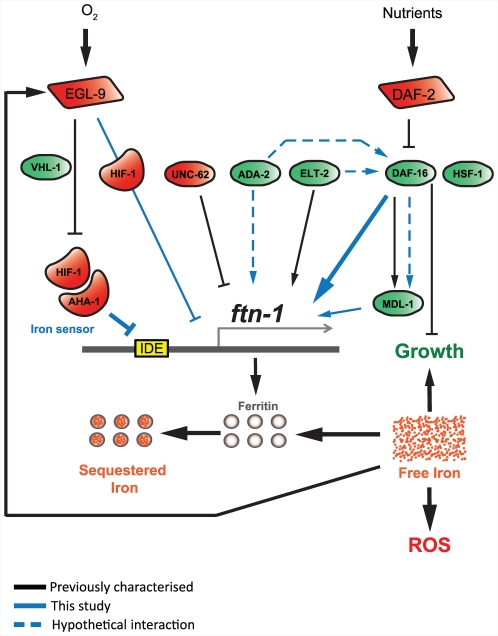
*ftn-1* expression is regulated by both the insulin/IGF-1 and hypoxia signaling pathways. This figure provides a diagrammatic representation of the gene regulatory networking controlling *ftn-1* expression. It includes previously established regulatory elements (black lines), newly established regulatory elements (blue lines) and new, hypothetical regulatory elements (dashed blue lines). We identified a positive regulatory role for the genes *mdl-1*, *hsf-1*, *ada-2* and *daf-16*. In the case of *mdl-1*, previous work suggests that this transcription factor acts downstream of DAF-16, but it is unclear whether this is true for *ftn-1* regulation (hence second line, dotted blue, from DAF-16 to MDL-1), or whether MDL-1 acts independently of DAF-16 in this case. Loss of *ada-2* or *elt-2* reduces *ftn-1* expression but we were unable to detect an effect of *ada-2* or *elt-2* RNAi in the absence of DAF-16. While this may be caused by a lack of sensitivity in our assay, it could also indicate that these factors may act together with DAF-16 or upstream of DAF-16 to regulate *ftn-1* expression. We found that *hif-1* and *aha-1* repress *ftn-1* expression and that *hif-1* is required for iron-dependent regulation of *ftn-1*, implying that HIF acts as an iron sensor in *C. elegans*. However, HIF-1 activity on *ftn-1* expression can be regulated through both *vhl-1*-dependent and independent pathways and our data shows that these pathways act antagonistically on *ftn-1* expression. The VHL-1-independent inhibition of *ftn-1* expression by EGL-9 could either involve activation of transcriptional repression by HIF-1 or (more parsimoniously) inhibition of transcriptional activation by HIF-1. The latter interpretation would suggest the presence of a co-regulator that turns HIF-1 into a transcriptional activator of *ftn-1*.

We find that the effects of loss of *egl-9* on *ftn-1* do not require *vhl-1* either. But in contrast to the other examples cited above, regulation of *ftn-1* by the *vhl-1*-dependent and independent pathways downstream of EGL-9 act antagonistically, the former repressing *ftn-1* expression and the latter activating it. Thus, our findings imply that EGL-9 not only represses HIF-1 activity by the well-characterized VHL-1-dependent pathway, but also modulates HIF-1 activity by an unknown mechanism (Figure 7). Prolyl hydroxylases are sensitive proteins capable of responding not only to iron and oxygen levels, but also to cues from metabolism [60] and to ROS [61], [62]. One or more of these may trigger the VHL-independent activity of EGL-9.

### Coordinate regulation of *ftn-1* by IIS and hypoxia signaling

Previous studies have suggested that hypoxia and IIS might act in concert to regulate gene expression. *daf-2* mutants are highly resistant to hypoxia [63] and microarray studies found an over-representation of genes containing hypoxia response elements (HRE) among IIS/DAF-16 regulated genes [26]. A study using murine embryonic fibroblasts found that the FOXO3a transcription factor inhibits HIF-1 mediated gene regulation [64]. We were therefore interested in investigating whether hypoxia signaling and IIS interact to regulate *ftn-1*.

One model for the joint regulation of *ftn-1* by *hif-1* and IIS/DAF-16 that we initially considered is that loss of *hif-1* activates DAF-16 which in turn activates *ftn-1*. DAF-16 is a stress-responsive transcription factor, so it seemed possible that stress caused by loss of HIF triggers a DAF-16-mediated cytoprotective response. There is evidence that loss of *hif-1* does indeed have this effect on DAF-16: *hif-1* mutants are long-lived and this lifespan extension has been shown to require *daf-16* [49]. However, several observations argue against the idea that DAF-16 mediates HIF-1 effects. Firstly, HIF-1 binds directly to the *ftn-1* promoter (Figure 4F). Secondly, the effects of loss of *hif-1* on *ftn-1* expression do not require *daf-16* (Figure 3E). Finally, loss of *hif-1* can further induce the expression of *Pftn-1::gfp* in *daf-2* mutants.

The fact that *daf-16* and *hif-1* have opposite effects on *ftn-1* expression bears consideration. Recent reports have found that HIF-1 overexpression extends lifespan [49], suggesting that HIF-1 activity has a similar effect to increased DAF-16 activity. Whether this occurs through the activation of a similar set of genes is unknown. Our finding that HIF-1 and DAF-16 can have opposing effects on gene expression suggests that the relationship between the two gene-sets is complex. Regulation of *ftn-1* by HIF-1 and DAF-16 could be a special case in which DAF-16 regulation occurs as part of a broad response to lower oxidative stress whereas HIF-1 acts as an iron sensor. Further work is required in order to establish whether antagonistic regulation by DAF-16 and HIF-1 is specific to *ftn-1* or whether it represents a more general pattern of gene regulation by the two pathways.

In summary, this study maps out a complex gene-regulatory network controlling expression of *ftn-1* and, by extension, iron homeostasis in the nematode *C. elegans*. This reveals the acute sensitivity of iron homeostasis to environmental conditions, allowing fine tuning of iron availability in the face of variability of factors that increase free iron (increased environmental iron, growth arrest, increased autophagy) and decrease it (reduced environmental iron, increased growth). Our results also underscore the value of *C. elegans* as a model system for understanding mammalian iron homeostasis, and the pathologies that can result from its breakdown.

## Materials and Methods

### Nematode culture and strains

Maintenance and culture of *C. elegans* was carried out as published [65], [66], [67]. The following strains were used: CB5602 *vhl-1(ok161)*, DR1563 *daf-2(e1370)*, DR1567 *daf-2(m577)*, GA300 *daf-16(mgDf50); daf-2(m577)*, GA633 *daf-2(m577); wuIs177 [Pftn-1::gfp lin-15(+)]*, GA636 *rrf-3(pk1426); daf-2(m577); wuIs177 [Pftn-1::gfp lin-15(+)]*, GA639 *daf-16(mgDf50); wuIs177 [Pftn-1::gfp lin-15(+)]*, GA640 *wuIs176 [Pftn-1::gfp lin-15(+)]*, GA641 *wuIs177 [Pftn-1::gfp lin-15(+)]*, GA642 *hif-1(ia4); wuIs177 [Pftn-1::gfp lin-15(+)]*, GA643 *daf-16(mgDf50); daf-2(m577)*; *wuIs177 [Pftn-1::gfp lin-15(+)]*, GA654 *unc-119(ed3) vhl-1(ok161) iaIs128[Phif-1::hif-1a::myc unc-119(+)]*, GA675 *xtEx79 [Δpes-10(+63)::GFP-his, pha-1(+)]*, GA676 *hif-1(ia4) xtEx79 [Δpes-10(+63)::GFP-his, pha-1(+)]*, GA688 *pha-1(e2123ts) xtEx79 [Δpes-10(+63)::GFP-his, pha-1(+)]*, GA688 *pha-1(e2123ts); hif-1(ia4) xtEx79 [Δpes-10(+63)::GFP-his, pha-1(+)]*, GA694 *wuIs176 [Pftn-1::gfp lin-15(+)] egl-9(sa307)]*, GA1200 *mdl-1(tm311)*, GA1203 *daf-2(e1370); mdl-1(tm311)*, GA1204 *daf-2(m577); mdl-1(tm311)*, GR1307 *daf-16(mgDf50)*, JT307 *egl-9(sa307)*, N2, PS3551 *hsf-1(sy441)*, UZ96 *pha-1(e2123ts) xtEx79 [Δpes-10(+63)::GFP-his, pha-1(+)]*, XA6900 *pha-1(e2123ts) qaEx6902 [Pftn-1(Δ63)::[Δpes-10::GFP-his, pha-1(+)]*, XA6902 *pha-1(e2123ts) qaEx6902 [Pftn-1::[Δpes-10::GFP-his, pha-1(+)]* and ZG31 *hif-1(ia4)*. ZG429 *unc-119(ed3) iaIs128[Phif-1::hif-1a::myc unc-119(+)]* Worms were maintained at 20°C unless otherwise indicated.

### Strain constructions

Multiple mutants were created using standard methodologies and the presence of genomic deletions was tested via PCR. Genotyping was carried out by lysis of parent animals using proteinase K (Sigma) and subsequent PCR using the following primers. For *daf-16(mgDf50)*: daf-16F1, gccactttattggaatttgagc; and daf-16R1, atcctcccatagaaggaccatt. For *hif-1(ia4)*: hif-1_ex_fwd1, gctcctcctactccacctttg, hif-1_ex_rev1, gtgacgagttgtgaatgcacc, hif-1_int_rev1.2, tcggcgatggtgtcttcagtc. For *rrf-3(pk1426)*: rrf-3_ex_fwd1, gagttcgcatcaagtttcac, rrf-3_ex_rev1, tgccttcgtacatttcaacc and rrf-3_int_rev2, ggtatttattgcttcctgccac. For *vhl-1(ok161)*: DA75, gctgtcaatcggagcactgtc, DA76, ttgctgaggtctctggggtc, and DA77, gttagctctgccacgaatacgatg. For *egl-9(sa307)*: DA117, acaaagacaggtgttgcgaatgag, DA118, ttgtagtgatccgagcccag, and DA119, gatgcttctgatgttcttggagg.

The *promoter::gfp* transgene of *ftn-1* was created using methods as previously described [68] and the transgenic strain was created by microinjection. The primers used for creation of the construct were: ftn-1.5'ex, tgcttactggttctgccgag, ftn-1.5'in, tgtagggtttgattgtggtttg, ftn-1.3'fus, agtcgacctgcaggcatgcaagctttgacgagctagagacatgac. Extrachromosomal arrays were integrated by X-ray irradiation.

### Fluorescence measurements

The method used to quantify GFP expression was adapted from one used in an earlier study [69]. Using a worm pick, samples of forty adult worms were transferred into the wells (V-shaped) of microtitre plates (Greiner). Fluorescence was then measured in a GeniosPlus plate reader (Tecan) at wavelengths appropriate for GFP (excitation: 495 nm; emission: 535 nm) using a fixed gain of 75. Quantification of GFP expression from transgenes with low level expression was carried out using a Leica DMRXA2 microscope using a GFP filter cube (excitation: 470/40 nm; emission: 525/50 nm), an Orca C10600 digital camera (Hamamatsu) and Volocity image analysis software (Improvision).

### RNAi library

The transcription factor RNAi library used for this project was generously provided by Dr. Weiqing Li (University of Washington). Similar libraries are now available commercially (geneservice.co.uk). Where RNAi robustly affected *ftn-1* expression levels, RNAi plasmid inserts were sequenced to confirm their identity using the primers JJM130 (gggaagggcgatcggtgcgggcc) and JJM131 (gcgcagcgagtcagtgagcgagg).

### qRT–PCR

RNA was isolated from 2-day old adults after three washes, which removed *E. coli* and L1 progeny from the sample. After RNA isolation cDNA was synthesized using SuperScript II reverse transcriptase (Invitrogen) using oligo dT (Invitrogen). qRT-PCR was carried out using Fast SYBR Green Master Mix (Applied Biosystems) and the 7900 HT Fast PCR system (Applied Biosystems). Normalization of transcript quantity was carried out using the geometric mean of three stably expressed reference genes Y45F10D.4, *pmp*-3, and *cdc-42* in order to control for cDNA input, as previously described [70]. The following primers were used for this assay. Y45F10D.4: DA90, gtcgcttcaaatcagttcagc, and DA91, gttcttgtcaagtgatccgaca. *pmp-3*: DA88, gttcccgtgttcatcactcat, and DA89, acaccgtcgagaagctgtaga. *cdc-42*: DA86, ctgctggacaggaagattacg, and DA87: ctcggacattctcgaatgaag. *ftn-1*: ftn-1_fwd_RT2, cggccgtcaataaacagattaacg, and ftn-1_rev_RT2 cacgctcctcatccgattgc.

qRT-PCR of ChIP DNA pools was carried out for the *nhr-57* promoter using DA130: cctcccgcgtctccacattcaatc and DA131: cagcgaggtctgggttttccg, the *nhr-57* 3′UTR using DA135: tggcacaagatatgacgaaagctg and DA136: ggcgagaaatttgttgtaggttgcc, and the *ftn-1* promoter using DA139: aacagctcacgtagccaatgataag and DA140: gcatcacatgagctgcccta.

### Statistical analysis

All results shown are the mean of at least three independent biological replicates and error bars represent the s.e.m. Statistical significance was calculated by two-way or one-way ANOVA of either raw values or log-transformed quantities, depending on circumstances.

### Chromatin immunoprecipitation

The protocol for chromatin immunoprecipitation was adapted from Mukhopdhyay et al. [71]. *C. elegans* cultures were grown for two generations in S-media with suspended OP50 at 20°C with constant shaking at 200 rpm. The worms were collected and washed four times in PBS buffer and then re-suspended in PBS containing 1% formaldehyde. Samples were then partially lysed using 8 strokes with a 1/3 turn in a 7 cm Dunce homogenizer and then incubated for 17 minutes with gentle mixing at room temperature. Crosslinking was stopped by addition of 200 µl 2.5 mol/L Glycine solution and 20 minutes further incubation at room temperature. After four washes in PBS containing protease inhibitor tablets (Complete, Roche), samples were flash frozen and stored at −80°C. After thawing, 2 mL of HLB buffer [50 mM HEPES-KOH, pH 7.5, 150 mM NaCl, 1 mM EDTA, 0.1% (wt/vol) sodium deoxycholate, 1% (vol/vol) Triton X-100, 0.1% (wt/vol) SDS and 1× Complete protease inhibitor] was added and sonication was carried out at 70% intensity for 7 bursts of 30 seconds in the Vibracell sonicator (Sonics). Protein quantity was estimated by Bradford assay (Biorad) and 2 mg were diluted into to 500 µl of in HLB buffer. Three 50 µl aliquots were removed at this point. DNA isolated from these samples was subsequently used as input controls. Samples were precleared for 1 h in prewashed salmon sperm DNA/protein-A agarose beads (Millipore) and then incubated overnight with 10 µl of anti-Myc Ab (9b11; Cell signalling). Samples were then incubated with prewashed salmon sperm DNA/protein-A agarose beads for 2 h. The beads were then washed twice in WB1 [50 mM HEPES-KOH, pH 7.5, 150 mM NaCl, 1 mM EDTA, 1% (wt/vol) sodium deoxycholate, 1% (vol/vol) Triton X-100, 0.1% (wt/vol) SDS and 1× Complete protease inhibitor], twice in WB2 [50 mM HEPES-KOH, pH 7.5, 1 M NaCl, 1 mM EDTA, 1% (wt/vol) sodium deoxycholate, 1% (vol/vol) Triton X-100, 0.1% (wt/vol) SDS and 1× Complete protease inhibitor] and once in WB3 [50 mM Tris-HCl, pH 8, 0.25 mM LiCl, 1 mM EDTA, 0.5% (vol/vol) NP-40 and 0.5% (wt/vol) sodium deoxycholate]. Crosslinking was reversed by addition of proteinase K solution [50 mM Tris-HCl, pH 8, 25 mM EDTA, 1.25% (wt/vol) SDS, 160 µg/ml proteinase K (Qiagen)] and incubation for 2 h at 45°C and overnight at 65°C. DNA was isolated by applying solution to Qiagen PCR purification columns (Qiagen).

## Supporting Information

Figure S1 (A) Worms carrying mutations in the genes *daf-16*, *mdl-1* and *hsf-1* as well as wild type worms were grown at 20°C until early adulthood. At this point, they were washed off the plates and transferred to plates containing 25 mM ferric ammonium citrate (FAC). Samples were collected for qRT-PCR after 18 h. (B) Synchronized L1 animals of these same strains were grown on either control RNAi cultures or treated with RNAi against *hif-1*. Samples were collected on the first day of adulthood for qRT-PCR. Statistical significance calculated by ANOVA. ***: p<0.001.
(EPS)

Figure S2 (A) Effect of addition of iron (25 mM FAC) on *ftn-1* transcript levels in wild type and *hif-1(ia4)* mutants. (B) Effect of addition of 0.1 mM bipyridyl (BP on *ftn-1* transcript levels in wild type and *hif-1(ia4)* mutants. Statistical significance calculated by ANOVA. ***: p<0.001.
(EPS)

Figure S3 (A) Effect of the *n586* allele of *egl-9* on expression of *ftn-1*. After two rounds of outcrossing, expression of *ftn-1* was quantified in *egl-9(n586)* as well as N2 control animals. (B) Effect of *egl-9* deletion on *Pftn-1::gfp expression* in *vhl-1(+)* and *vhl-1(ok161)* animals. (C) Effect of *egl-9* deletion on *ftn-1* transcript levels in *vhl-1(+)* and *vhl-1(ok161)* animals. Samples were collected at day 1 of adulthood. (D) Effect of *egl-9* deletion on *ftn-1* transcript levels in *hif-1(+)* and *hif-1(ia4)* animals. Statistical significance calculated by ANOVA. **: p<0.01, ***: p<0.001.
(EPS)

Table S1 RNAi of a large number of genes altered expression of *Pftn-1::gfp* in the primary screen. Table S1 contains a list of RNAi treatments that reduced expression of the transgene by at least 20% and shows which of these effect were confirmed first using an alternative strain and then using qRT-PCR of the *ftn-1* transcript.
(DOCX)

Table S2 Table S2 contains a list of RNAi treatments found to increase expression of *Pftn-1::gfp* by at least 20% in the primary screen.
(DOCX)
