# Supplementary material for: Insulin/IGF-1 and Hypoxia Signaling Act in Concert to Regulate Iron Homeostasis in Caenorhabditis elegans
Source: PLoS Genet. 2012 Mar 1;8(3):e1002498. doi: 10.1371/journal.pgen.1002498 (PMC3291539; doi:10.1371/journal.pgen.1002498)
Supplement: Table S1 — RNAi of a large number of genes altered expression of Pftn-1::gfp in the primary screen. Table S1 contains a list of RNAi treatments that reduced expression of the transgene by at least 20% and shows which of these effect were confirmed first using an alternative strain and then using qRT-PCR of the ftn-1 transcript. (DOCX) [file pgen.1002498.s004.docx]

Table S1: Genes for which RNAi reduces *Pftn-1::gfp* expression

| **Gene** | **% of control RNAi** | **Confirmed using:** | | **Gene description** |
| --- | --- | --- | --- | --- |
|  |  | *Pftn-1::gfp* | *ftn-1* transcript |  |
| *daf-16* | 19.7 | * | ** | FoxO transcription factor |
| *klf-1* | 23.6 | * |  | Krueppel-like factor |
| *ast-1* | 26.8 |  |  | Determinant of DA neuron fate ETS-box |
| *hsf-1* | 31.5 | * | ** | Heat shock factor |
| *ada-2* | 37.5 | * | ** | Ada2 homolog |
| *nhr-116* | 45.6 |  |  | Nuclear hormone receptor |
| B0336.3 | 53.2 |  |  | Contains RNA recognition motif |
| *dve-1* | 55.9 |  |  | *Drosophila* DVE homolog |
| *egr-1* | 56.1 | * |  | Homolog of mammalian MTA proteins |
| *psa-1* | 57.2 |  |  | Component of the SWI/SNF complex |
| *hlh-30* | 58.5 |  |  | Helix loop helix factor |
| *blmp-1* | 58.8 | * |  | Zinc finger protein |
| *pbrm-1* | 60.6 | * |  | Polybromo 1 ortholog |
| *mdl-1* | 64.3 | * | ** | Similar to vertebrate MAD |
| K10B3.5 | 64.5 |  |  | Nematode specific protein |
| *nhr-66* | 67.4 |  |  | Nuclear hormone receptor |
| *nhr-75* | 68.4 |  |  | Nuclear hormone receptor |
| T07C12.11 | 68.5 | * |  | Nematode specific protein |
| *nhr-34* | 69.6 |  |  | Nuclear hormone receptor |
| *fkh-6* | 70.3 |  |  | Forkhead transcription factor |
| *elt-2* | 73.0 | * | ** | GATA-type transcription factor |
| *pqm-1* | 74.5 | * |  | C2H2-type zinc finger and leucine zipper-containing protein |
| F44E2.7 | 74.6 |  |  |  |
| *nhr-36* | 75.8 |  |  | Nuclear hormone receptor |
| *lin-29* | 76.4 |  |  | Zinc finger protein |
| *jun-1* | 77.4 |  |  | JUN homologue |
| *lir-1* | 77.6 |  |  | Zinc finger protein |
| *nhr-80* | 78.1 |  |  | Nuclear hormone receptor |
| *lin-40* | 78.4 |  |  | Zinc finger protein |
| *cdc-14* | 79.8 |  |  | Cdc14p homologue |

* RNAi of genes that resulted in a greater than 20% decrease in GFP expression from the GA636 strain was investigated further. The effect of these RNAi clones was confirmed using the GA633 strain: *wuIs177[Pftn-1::gfp] daf-2(m577)] .* This secondary test was carried out after confirming the identity of RNAi clones. Plasmids were then retransformed into HT115 bacteria.

** To exclude transgene-specific effect, the effect of RNAi on the *ftn-1* transcript itself was tested by qRT-PCR (See Figure 2A).
