# Supplementary material for: Insulin/IGF-1 and Hypoxia Signaling Act in Concert to Regulate Iron Homeostasis in Caenorhabditis elegans
Source: PLoS Genet. 2012 Mar 1;8(3):e1002498. doi: 10.1371/journal.pgen.1002498 (PMC3291539; doi:10.1371/journal.pgen.1002498)
Supplement: Table S2 — contains a list of RNAi treatments found to increase expression of Pftn-1::gfp by at least 20% in the primary screen. (DOCX) [file pgen.1002498.s005.docx]

Table S2: Genes for which RNAi increases *Pftn-1::gfp* expression

| **Gene** | **Mean % of control** | **Description** |
| --- | --- | --- |
|  |  |  |
| *aha-1* | 317.1 | Ortholog of human ARNT |
| *hif-1* | 309.5 | Hypoxia –inducible factor α homolog |
| *unc-62* | 258.2 | Meis-class homeodomain protein |
| *irx-1* | 195.3 | Homeodomain protein |
| *fkh-10* | 182.3 | Forkhead transcription factor |
| *let-607* | 177.9 | CREB/ATF family transcription factor |
| *ceh-60* | 177.8 | PBX family of homeodomain transcription factors |
| *ceh-18* | 166.1 | POU-class homeodomain transcription factor |
| *nhr-165* | 149.3 | Nuclear hormone receptor |
| *gei-17* | 148.6 | Zinc finger protein |
| *pqn-21* | 148.5 | Contains Q/N-rich domain |
| *repo-1* | 146.2 | Putative splicing factor |
| *fkh-9* | 142.8 | Forkhead transcription factor |
| *mep-1* | 142.0 | Zinc finger protein |
| *ztf-23* | 139.7 | Zinc finger protein |
| Y60A9.3 | 137.1 | Zinc finger protein |
| *pqn-75* | 136.9 | Contains Q/N-rich domain |
| *ztf-22* | 133.0 | Zinc finger protein |
| *cey-4* | 131.9 | Y-box containing protein |
| *ceh-52* | 131.5 | eHomeobox protein |
| *tbx-36* | 131.3 | T-box factor |
| F47G4.6 | 130.7 |  |
| *ehn-3* | 130.3 | Zinc finger protein |
| *nhr-7* | 128.3 | Nuclear hormone receptor |
| *nhr-265* | 127.2 | Nuclear hormone receptor |
| *nhr-141* | 127.2 | Nuclear hormone receptor |
| *klf-3* | 126.1 | Krueppel -like factor |
| *isw-1* | 121.3 | Homolog of chromatin remodelling ATPase ISW1 |

Only RNAi treatments that led to >20% induction are included.
